# Helminthic Infections Rates and Malaria in HIV-Infected Pregnant Women on Anti-Retroviral Therapy in Rwanda

**DOI:** 10.1371/journal.pntd.0002380

**Published:** 2013-08-15

**Authors:** Emil Ivan, Nigel J. Crowther, Eugene Mutimura, Lawrence Obado Osuwat, Saskia Janssen, Martin P. Grobusch

**Affiliations:** 1 Kigali Health Institute Department of Biomedical Laboratory Sciences, Kigali, Rwanda; 2 Rwanda Biomedical Centre National Reference Laboratory, Department of Laboratory Network, Kigali, Rwanda; 3 Department of Chemical Pathology, University of the Witwatersrand Medical School, National Health Laboratory Services, Parktown, Johannesburg, South Africa; 4 Women Equity in Access to Care and Treatment (WE-ACTx), Kigali, Rwanda and Regional Alliance for Sustainable Development (RASD Rwanda), Kigali, Rwanda; 5 Department of Medical Laboratory Science, School of Health Sciences, Mount Kenya University, Kigali, Rwanda; 6 Institute of Tropical Medicine, University of Tübingen, Tübingen, Germany; 7 Center of Tropical Medicine and Travel Medicine, Division of Internal Medicine, Academic Medical Center, University of Amsterdam, Amsterdam, The Netherlands; Rosetta Genomics, Israel

## Abstract

**Background:**

Within sub-Saharan Africa, helminth and malaria infections cause considerable morbidity in HIV-positive pregnant women and their offspring. Helminth infections are also associated with a higher risk of mother-to-child HIV transmission. The aim of this study was to determine the prevalence of, and the protective and risk factors for helminth and malaria infections in pregnant HIV-positive Rwandan women receiving anti-retroviral therapy (ART).

**Methodology and principle findings:**

Pregnant females (n = 980) were recruited from health centres in rural and peri-urban locations in the central and eastern provinces of Rwanda. Helminth infection was diagnosed using the Kato Katz method whilst the presence of *Plasmodium falciparum* was identified from blood smears. The prevalence of helminth infections was 34.3%; of malaria 13.3%, and of co-infections 6.6%. Helminth infections were more common in rural (43.1%) than peri-urban (18.0%; p<0.0005) sites. A CD4 count ≤350 cells/mm^3^ was associated with a higher risk of helminth infections (odds ratio, 3.39; 95% CIs, 2.16–5.33; p<0.0005) and malaria (3.37 [2.11–5.38]; p<0.0005) whilst helminth infection was a risk factor for malaria infection and vice versa. Education and employment reduced the risk of all types of infection whilst hand washing protected against helminth infection (0.29 [0.19–0.46]; p<0.0005);). The TDF-3TC-NVP (3.47 [2.21–5.45]; p<0.0005), D4T-3TC-NVP (2.47 [1.27–4.80]; p<0.05) and AZT-NVP (2.60 [1.33–5.08]; p<0.05) regimens each yielded higher helminth infection rates than the AZT-3TC-NVP regimen. Anti-retroviral therapy had no effect on the risk of malaria.

**Conclusion/significance:**

HIV-positive pregnant women would benefit from the scaling up of de-worming programs alongside health education and hygiene interventions. The differential effect of certain ART combinations (as observed here most strongly with AZT-3TC-NVP) possibly protecting against helminth infection warrants further investigation.

## Author Summary

There is an overlap in the worldwide distribution of intestinal worms (helminths), malaria and HIV. Co-infections with helminth and malaria parasites cause a significant problem in the host, particularly in the presence of HIV infection. The aim of this study was to assess the prevalence of intestinal worm and malaria infection and co-infections and the associated risk factors among HIV-positive pregnant women that attended rural and peri-urban health centers in Rwanda. Our findings indicate that intestinal worms were more common among HIV-infected pregnant women in the rural than peri-urban settings. HIV-positive pregnant women who had lower CD4 cell counts were more at risk of being infected by intestinal worms and malaria. Malaria also increased the risk of being infected by intestinal worms and vice versa. Socio-economic factors such as lack of education and unemployment were among the risk factors for intestinal worm infections and malaria. Hand washing was found to reduce the risk for worm infections; whilst one particular ART combination (AZT-3TC-NVP) led to a reduced rate of helminth infections when compared to others.

## Introduction

Globally, the most common nematode species that cause soil-transmitted helminthic diseases are *Ascaris lumbricoides*, *Trichuris trichiura*, and the hookworm species *Necator americanus and Ancylostoma duodenale* [1], [2], [3]. Although morbidity due to helminths can be controlled by delivering preventive chemotherapy with antihelminthic medicines, elimination and finally eradication will not be achieved until affected populations have access to effective sanitation, sewage treatment and waste disposal; which remains a common problem in most rural African settings. In most of sub-Saharan Africa, the health burden of helminthic disease is enormous [4]. Co-infections with malaria and HIV are numerous and important causes of morbidity and mortality. Combating co-infections has been identified as an important public health goal [5]. Important areas of current research interests are the effects of helminth infections on immune regulation and their possible consequences for susceptibility to other infections and immunologically mediated conditions such as allergy and autoimmune diseases [6].

The immunological interplay between helminth infections and HIV is complex, and there are different hypotheses on the influence of the infections on each other; the most important being the Th2 bias induced by helminth infections, suppressing Th1 responses specific to HIV; thus leading to more rapid HIV progression [7], [8]. HIV acquisition was positively correlated with female urogenital schistosomiasis [9] but in contrast, a randomized controlled trial (RCT) showed no benefit of deworming on prevention from mother to child transmission of HIV [10]. A few systematic reviews have been published describing the effect of anti-helminthic treatment on markers for HIV disease progression showing inconsistently beneficial effects of anti-helminthic treatment; lower increases in HIV viral loads and increases in CD4 counts have been reported [11]–[13]. However, results from a recent RCT did not suggest a beneficial role of empric deworming to delay HIV progression [14].

Risk factors for helminth infections depend on the route of transmission and the life cycles of the various helminth species; they are usually related to hygiene and sanitation [15]. The geographical distribution of helminth infections is largely influenced by several environmental factors such as climate and presence of stagnant water bodies [16]. In the absence of vaccinations, the only currently recommended public health intervention for soil transmitted helminths is regular mass de-worming, particularly for high risk groups; backed up by facilitating access to clean water, improved sanitation and health education [17].

Pregnancy may increase susceptibility to helminths, but this is uncertain. A recent study from Gabon showed increased prevalence in pregnancy [18], but a study from Thailand found no association [19]. Susceptibility and clinical outcomes are further complicated by co-infection with HIV and malaria. Malaria in pregnancy due to *Plasmodium falciparum*, combined with helminthic infections in HIV-positive women is of great concern from a public health perspective [20], [21]. Control of *P. falciparum* infection by intermittent preventive treatment and use of insecticide-treated bed nets is of high importance especially in primigravidae [22], [23]. HIV-infected women tend to experience faster CD4 decline during and after pregnancy [24], and could therefore be even more susceptible to helminth infection and malaria.

This prospective cohort study was designed to assess the major risk factors for helminth and malaria co-infection in HIV-positive pregnant women who participated in an early anti-retroviral therapy (ART) initiation program for the prevention of mother-to-child transmission in Rwanda. We describe the baseline prevalence of malaria and helminth infection in HIV-infected pregnant women on ART, and assess the factors that may increase or decrease rates of both infections.

## Methods

### Ethics Statement

Ethical approval was obtained from the Rwanda National Ethics Committee and the Ethics Committee (Human Research) of the University of the Witwatersrand Medical School, Johannesburg, South Africa. All subjects provided written informed consent at enrolment. Subjects who could only provide oral consent were asked to give thumb marks using indelible ink on the consent form, in case they were illiterate; in accordance with the IRB oral standard ethical consent guidelines of the two ethical committees and in accordance with Helsinki declarations.

### Study Population and Procedures

The study participants were recruited amongst women accessing antenatal care and ART services at rural and peri-urban health centers in the central and eastern provinces of Rwanda, between 02 January 2010 and 29 February 2011. After giving written informed consent, women in the second trimester of pregnancy were enrolled at their fourth, fifth or sixth month of gestation. Additionally, women were enrolled if they lived within walking distance from the study areas, and if they planned to deliver at the registered study health center. Enrolment criteria were HIV infection; pregnancy (in the second trimester); use of ART; and willingness to provide three stool samples on consecutive days. Women were excluded if they were diagnosed with tuberculosis, or if they had taken any anti-helminthic drugs at any time point prior to entry into the study. Those who had been enrolled in other research projects during the study period were also excluded. Participants provided blood and stool samples before being treated in accordance with the study protocol.

### Collection of Demographic, Socio-economic and Pregnancy-Related Data

On enrolment, participants were interviewed to obtain demographic and socio-economic information pertaining to the relevant environmental risk factors for helminth infections and malaria. Subjects were asked whether they had attended school, whether they were employed, their source of water (river or piped), if they wore shoes, whether they washed their hands after using the toilet, and whether they used dietary supplements. Study participants were also asked about their number of previous pregnancies.

### Diagnosis and Treatment of Infections

Intestinal helminths were identified by the Kato Katz method [22], [23]. Three Kato Katz slides were prepared from each stool sample, and then examined within 30 minutes for hookworm species. The same specimens were again examined the following day for ova of other soil transmitted helminths with the formol ether concentration method. Eggs per gram (EPG) of stool were calculated by taking the mean of the mean values obtained for each of the three stool samples. In all study women asymptomatic parasitaemia was determined. *Plasmodium falciparum* was identified by light microscopic examination of Giemsa stained thick and thin blood smears, and screening for malaria was also performed by detection of the *P. falciparum* histidine rich protein 2 (HRP-2) antigen using a rapid diagnostic test kit (Biotec Laboratories Ltd., UK). In the case of discordant results, expert light microscopy results were considered as gold standard. Participants received treatment for helminth infections and malaria in accordance with the study protocol. Artemether/lumefantrine (120/20 mg) was given as standard falciparum malaria treatment, administered orally in four doses for three days. Deworming was performed every 12 weeks with 400 mg albendazole given to women with helminth infections only/not to those negative in the ‘targeted treatment’arm; whereas all women received 400 mg albendazole irrespective of infection status in the ‘untargeted treatment’arm.

Women received nevirapine for prevention of mother-to-child HIV transmission and subsequent combination ART, irrespective of CD4 cell levels in accordance with the latest (2010) Rwandan Ministry of Health treatment guidelines [25].

### Statistical Analysis

Data analysis was performed using Stata version 11.0 (College Station, TX, USA) and Statistica version 9.1 (StatSoft, Tulsa, OK, USA). Data that was not normally distributed was log transformed to normality before analysis.

The prevalence of helminth, malaria and helminth-malaria co-infections were determined in population sub-groups e.g. in employed and unemployed subjects, and differences between the groups were assessed using the χ^2^ test.

Backward, stepwise multiple logistic regression analysis was used to determine the principal factors associated with helminth and malaria infections and helminth-malaria co-infections. Models were constructed for infection with each individual helminth species, i.e. *A. lumbricoides*, *T. trichiura* and hookworms, and a combined model for infection with any helminth species. The independent variables included in the initial logistic regression models were, for helminth infections: location, month of year, ART, gravidity, education, employment, water source, use of shoes, hand washing, dietary supplement use, HIV viral load, presence/absence of malaria, age, height, gestational age and CD4 counts. With malaria as the dependent, dichotomous variable, the same list of independent variables was used. The presence/absence of helminth infection was included as an additional independent variable whilst presence/absence of malaria was removed. With co-infection (helminth-malaria infection) as the dependent variable, both malaria and helminth presence/absence were removed from the model. In all the logistic regression models, the independent variable with the highest p-value was removed at iteration until only variables with a p<0.05 were left in the model.

Backward, stepwise multiple regression analysis was used to identify the principal determinants of fecal helminth egg counts and blood hemoglobin levels. Univariate analyses were initially performed, and any variable with p<0.50 was included as an independent variable in the multiple regression models. The same procedure as described for the logistic regression models was then followed.

A sample size calculation was not performed for this study. N was chosen based on logistical factors, taking into account future follow-up studies. However, if one performs a post-study sample size calculation based on the logistic regression model for identifying the principal determinants of helminth infection and using the equation N = (10*k)/p [26], where k is the number of co-variables (k = 18) and p is the frequency of helminth infections (p = 0.34), we obtain a minimum N of 529, which is far below the actual N of 980.

## Results

### Prevalence of Helminth Infections and Malaria

The data in Table 1 show that infection with any helminth species (in the presence or absence of malaria) occured in 336 participants (34.3% of the population investigated), being significantly (p<0.0005) more common in rural than in peri-urban communities. Infection with helminths in the absence of malaria showed a similar trend, occurring in 36.5% of rural, and in 11.3% of peri-urban subjects (p<0.0005). Infection with each of the three helminth species also occured more often in the rural than the peri-urban population, with *A. lumbricoides* being the most commonest. The presence of a malaria infection (in the presence or absence of helminths) was more frequent in peri-urban than in rural subjects (p<0.05). This trend was mirrored by malaria-only infections, with a prevalence of 4.39% in rural and 10.7% in peri-urban females (p<0.0005). The prevalence of helminth-malaria co-infection was similar in both environments (Table 1).

**Table 1:** Prevalence of helminth and malarial infections in rural and peri-urban populations.

Variables		Rural	Peri-urban	Combined
***n***		635	345	980
***Helminth infection***		43.1 (39.3–47.0)	18.0 (13.9–22.0)***	34.3 (31.3–37.3)
***Specific species:***	***Ascaris lumbricoides***	26.1 (22.7–29.6)	11.0 (7.69–14.3)***	20.8 (18.3–23.4)
	***Trichuris trichiura***	8.66 (6.47–10.8)	4.35 (2.18–6.51)**	7.14 (5.53–8.76)
	***Hookworm***	8.35 (6.19–10.5)	2.61 (0.92–4.30)***	6.33 (4.80–7.85)
***Malaria infection***		11.0 (8.58–13.5)	17.4 (13.4–21.4)*	13.3 (11.1–15.4)
***Helminth-malaria co-infection***		6.61 (4.68–8.55)	6.67 (4.02–9.31)	6.63 (5.07–8.19)

All data expressed as percentage (95% confidence intervals);

* p<0.05,

** p<0.005,

*** p<0.0005 vs rural.

### Prevalence of Helminth Infections and Malaria in Different Population Sub-Groups

The prevalence of helminth, malaria and helminth-malaria co-infections were calculated for different population sub-groups (Table 2). Helminth infections of any type, asymptomatic malaria or co-infections were all less prevalent in subjects receiving AZT-3TC-NVP when compared to those taking d4T-3TC-NVP (p<0.005). Treatment with AZT-3TC-NVP was also associated with a lower prevalence of malaria or co-infection when compared to AZT-NVP therapy, and a lower prevalence of co-infection compared to TDF-3TC-NVP. The latter therapy was associated with a lower prevalence of asymptomatic malaria compared to subjects receiving AZT-NVP.

**Table 2:** Prevalence of helminth and malarial infections in relation to various risk factors.

Variables		*n*	Helminth (%)	Malaria (%)	Co-infection (%)
***ART:***	***AZT-3TC-NVP NNNNNVP***	299	27.4^**^	6.35^**^ †††	2.67^*^ †††
	***AZT-NVP***	126	32.5	24.6^**^	14.3^**^
	***d4T-3TC-NVP***	461	39.7	14.5	6.72
	***TDF-3TC-NVP***	94	31.9	13.8†	8.51‡
***Months:***	***Jan–Feb***	262	27.9	24.4	12.2
	***Mar–May***	718	36.6^*^	9.19^***^	4.60^***^
***Age:***	***≤30 years***	545	37.4	10.3	5.50
	***>30 years***	435	30.3^*^	17.0^**^	8.05
***Gravidity:***	***1***	225	42.2	16.0	10.2
	***2–5***	755	31.9^**^	12.4	5.56^*^
***Gestation:***	***4 months***	505	33.5	22.0	11.3
	***5–6 months***	476	35.2	4.00^***^	1.68^***^
***Education:***	***Some***	480	21.6	10.6	2.80
	***None***	500	47.5^***^	16.0^*^	10.6^***^
***Employment:***	***Yes***	181	28.3	10.9	3.63
	***No***	799	60.8^***^	23.8^***^	19.9^***^
***Water:***	***Piped***	215	13.0	16.7	3.26
	***River***	765	40.3^***^	12.3	7.58^*^
***Shoe wearing:***	***Yes***	380	30.5	16.3	8.16
	***No***	600	36.7^*^	11.3^*^	5.67
***Hand washing:***	***Yes***	695	29.3	17.7	8.63
	***No***	285	46.3^***^	2.46^***^	1.75^***^
***Diet supplements:***	***Yes***	281	28.1	21.3	11.7
	***No***	699	36.8^*^	10.0^***^	4.58^***^
***Viral load:***	***Detectable***	90	70.0	17.8	14.4
	***Not detectable***	890	30.7^***^	12.8	5.84^**^
***CD4:***	***≤350 cells/mm^2^***	209	62.7	27.7	20.6
	***>350 cells/mm^2^***	771	26.6^***^	9.34^***^	2.85^***^
***Malaria:***	***Present***	130	50.0	-	-
	***Absent***	850	31.9^***^	-	-
***Helminths:***	***Present***	336	-	19.3	-
	***Absent***	644	-	10.1^***^	-

All data expressed as percentage; *p<0.05, **p<0.005, ***p<0.0005 vs other sub-group of same variable.

For ART: *p<0.05, **p<0.005 vs d4T-3TC-NVP;

† p<0.05,

††† p<0.0005 vs AZT-NVP;

‡ p<0.05 vs AZT-3TC-NVP.

A number of factors had the opposite effect on helminth infection compared to malaria or co-infection rates. Thus, helminth infections were more common, but malaria and consequently co-infections less common in females who were tested in March–May compared to those tested in January or February. This same pattern was observed for females who did not wear shoes compared to those who did, and in females who did not regularly wash their hands or take dietary supplements when compared to those that did (Table 2).

Pregnant females who were older than 30 years at testing had a lower prevalence of helminth infections but higher levels of asymptomatic malaria and co-infections than those females who were 30 years or younger. Primigravidae had higher prevalences for all three infection types compared to females who had more than one previous pregnancy, whilst females who presented for testing at an earlier stage of their pregnancy (4 months) had higher prevalence levels of malaria and co-infections compared to those at a later stage of pregnancy (5–6 months) (Table 2). If this latter group was divided into 5 and 6 months of gestation, the prevalence of malaria was not significantly different between them (4.20% vs 3.52% respectively).

Study participants who were unemployed and subjects with no formal education had a higher prevalence of helminth infections, malaria and co-infections compared to subjects with employment and the uneducated, respectively. Women who used river surface rather than piped water had a higher prevalence of both helminth infection and co-infection but a lower prevalence of malaria, although this last comparison did not reach statistical significance (p = 0.09). A detectable viral load and a CD4 count ≤350 cells/mm^3^ were both associated with higher levels of all infections. Subjects with asymptomatic malaria had a higher prevalence of helminth infections, and vice versa (see Table 2).

### Identification of Risk and Protective Factors for Helminth Infections

Table 3 depicts the results of multiple logistic regression analyses to identify risk and protective factors for helminth infections. With regard to ART, the d4T-3TC-NVP regimen groups exhibited higher prevalences of infection with *A. lumbricoides* and hookworm compared to AZT-3TC-NVP. The same applied with the AZT-NVP and TDF-3TC-NVP regimens regarding *T. trichiura* prevalence when compared to the AZT-3TC-NVP therapy.

**Table 3:** Multiple logistic regression analyses to identify risk factors for helminth infections.

Variables		Odds ratios for *Ascaris* infection	Odds ratios for *Trichuris* infection	Odds ratios for hookworm infection	Odds ratio for any helminth infection
***ART*** †	***d4T-3TC-NVP***	2.59 (1.79–3.75)***	-	2.19 (1.27–3.79)**	3.47 (2.21–5.45)***
	***AZT-NVP***	-	4.65 (2.41–8.96)***	-	2.60 (1.33–5.08)*
	***TDF-3TC-NVP***	-	3.57 (1.69–7.57)**	-	2.47 (1.27–4.80)*
***Months:***	***Jan–Feb vs Mar–May***	-	-	0.32 (0.13–0.77)*	-
***Age:***	***>30 vs ≤30 yrs***	-	-	-	0.66 (0.47–0.94)*
***Gravidity:***	***>1 vs 1***	-	-	-	0.59 (0.39–0.87)*
***Location:***	***Periurban vs Rural***	0.52 (0.33–0.82)**	-	0.32 (0.15–0.66)**	0.41 (0.27–0.62)***
***Education:***	***Yes vs No***	0.41 (0.28–0.59)***	-	-	0.39 (0.28–0.55)***
***Employment:***	***Yes vs No***	0.47 (0.31–0.73)**	0.14 (0.08–0.27)***	-	0.23 (0.15–0.36)***
***Water:***	***Piped vs River***	0.30 (0.16–0.53)***	-	-	0.23 (0.14–0.38)***
***Hand washing:***	***Yes vs No***	0.52 (0.33–0.80)**	0.20 (0.10–0.40)***	-	0.29 (0.19–0.46)***
***Detectable viral load:***	***Yes vs No***	1.95 (1.11–3.42)*	-		2.42 (1.29–4.55)*
***CD4:***	***≤350 vs >350 cells/mm^3^***	2.12 (1.38–3.23)**	2.27 (1.32–3.90)**	3.03 (1.75–5.26)***	3.39 (2.16–5.33)***
***Malaria:***	***Infected vs Not***	-	-	-	2.13 (1.27–3.59)**

† as compared to AZT-3TC-NVP;

data are odds ratios (95% confidence intervals);

odds ratios for ARTs are in comparison to therapy with AZT-3TC-NVP;

odds ratios are not given for variables that had no significant effect and were removed from regression model;

the following variables did not significantly affect risk for any of the above infections: gestational age, wearing shoes, use of dietary supplements and height;

* p<0.05,

** p<0.005,

*** p<0.0005.

There were lower rates of hookworm infestation in subjects who were screened for infections during January and February compared to those screened later in the year, whilst subjects who were older than 30 years, or who were multigravid had a lower risk of any helminth infection when compared, respectively, to those 30 and younger, or primigravidae.

Subjects who were residents of a peri-urban location had a lower risk of *A. lumbricoides*, hookworm or any helminth infection in comparison to those from a rural environment. Educated study participants and those who used piped water were at a lower risk of *A. lumbricoides* or any helminth infection when compared, respectively, to subjects with no formal education and who used river water. Furthermore, pregnant women who were employed or who regularly washed their hands were at a lower risk for *A. lumbricoides*, *T. trichiura* or any helminth infection in comparison to subjects who, were employed or did not wash their hands regularly, respectively.

Pregnant females who had a detectable viral load when compared to those who did not, were at a higher risk for *A. lumbricoides*, and subjects with a CD4 count at or below 350 cells/mm^3^ were at a higher risk for all kinds of helminth infections when compared to those with CD4 counts above 350 cells/mm^3^. The presence of malaria was associated with a higher risk of any helminth infection.

### Identification of Risk and Protective Factors for Malaria and Co-Infections

The risk for malaria was higher in the months of January and February than from March to May (Table 4). Risk was also higher in older females but lower in those in the third trimester. This latter trend was also mirrored by risk for co-infection. Co-infection risk was also reduced in subjects with some formal education and in those with employment. Pregnant females who used piped rather than river surface water had a higher risk of malaria. Helminth infection was associated with a higher risk for malaria, whilst low CD4 counts were linked to a higher risk of malaria and co-infection. Interestingly, and being difficult to interpret, women who regularly washed their hands had a higher risk of both malaria and co-infections.

**Table 4:** Multiple logistic regression analyses to identify risk factors for malaria infection and helminth-malaria co-infection.

Variables		Odds ratios for malaria infection	Odds ratios for helminth-malaria co-infection
***Months:***	***Jan–Feb vs Mar–May***	1.70 (1.08–2.68)*	-
***Age:***	***>30 vs ≤30 years***	1.76 (1.15–2.69)*	-
***Gestation:***	***5–6 vs 4 months***	0.17 (0.10–0.29)***	0.16 (0.07–0.35)***
***Education:***	***Yes vs No***	-	0.32 (0.17–0.63)**
***Employment:***	***Yes vs No***	-	0.26 (0.14–0.49)***
***Water:***	***Piped vs River***	1.76 (1.04–2.97)*	-
***Hand washing:***	***Yes vs No***	5.81 (2.51–13.5)***	2.96 (1.07–8.19)*
***Helminth infection:***	***Yes vs No***	2.42 (1.51–3.89)***	-
***CD4:***	***≤350 vs >350 cells/mm^3^***	3.37 (2.11–5.38)***	7.13 (3.95–12.9)***

Data are odds ratios (95% confidence intervals); odds ratios are not given for variables that had no significant effect and were removed from regression model; the following variables did not significantly affect risk for any of the above infections: location, ART, viral load, gravidity, wearing shoes, use of dietary supplements and height;

* p<0.05,

** p<0.005,

*** p<0.0005.

### Identification of the Principal Determinants of Fecal Helminth Egg Count and Hemoglobin Level

Backward, stepwise multiple regression analysis demonstrated that fecal helminth egg counts were highest in females who were multigravid; who did not wear shoes and who had low CD4 counts (Table 5). Hemoglobin levels were lowest in females who had helminth or malaria infections, who had low CD4 counts and who had a higher (5 or 6 months compared to 4 months) gestational age.

**Table 5:** Multiple regression models for determinants of helminth egg count and hemoglobin level.

Model number	Dependent variable	Independent variables	Beta value (p value)	R for model (p-value)
***1***	Egg count (log)	Gravidity^a^	0.17 (0.001)	0.28 (<0.0005)
		Use of shoes^b^	−0.13 (0.01)	
		CD4 count (log)	−0.54 (0.03)	
***2***	Hemoglobin level	Helminth^c^	−0.67 (<0.0005)	0.34 (<0.0005)
		Malaria^c^	−0.31 (0.01)	
		CD4 count (log)	1.37 (<0.0005)	
		Gestational age^d^	−0.19 (0.02)	

a Gravidity coding: primigravida - 1, multigravida - 2;

b Coding for use of shoes: wear shoes - 1, do not wear shoes - 0;

c Coding for helminth or malaria: infected - 1, no infection - 0;

d Coding for gestational age: 4 months - 1, 5 or 6 months – 2.

## Discussion

In this study population, we determined the prevalence and identified protective and risk factors of helminth, malaria and co-infections in HIV-infected pregnant women on ART in Rwanda. We found that helminth infection was more prevalent in rural than peri-urban settings. Poor education and unemployment were risk factors for both helminth and *P. falciparum* infection, whilst hand washing protected against worm infections. HIV treatment with AZT-3TC-NVP was associated with a lower prevalence of helminth infections. A CD4 count ≤350 cells/mm^3^ was associated with higher levels of all infections. Multiple linear regression analysis demonstrated that helminth egg counts (EPG) were highest in females who were multigravid and hemoglobin levels were lowest in females who had helminth or malaria infections.

The prevalence of helminth infection was higher among the rural than peri-urban populations. Whilst we did not notice general differences between women recruited at the various health centers, this is best explained by variations in lifestyle between both settings. The most prevalent species were *A. lumbricoides* followed by *T. trichiura* and hook worm species (*A. duodenale and N. americanus*). This is in agreement with previous findings from the same location [21]. Our results are further supported by findings from an earlier study in the region which indicated that *A. lumbricoides* and *T. trichiura* were more commonly found in Rwanda and Burundi than in most other East African countries [27]. Our findings show lower prevalence levels for malaria and malaria-helminth co-infection than previously reported for pregnant females in Ghana but higher rates of helminth infections [28]. A study in Uganda [29] reported that the prevalence of helminth infection among pregnant women was 68% and malaria was 11%; however, only 12% of the women were HIV infected. These results indicate (not surprisingly) that there are varying prevalence levels of helminth infection during pregnancy across East African populations. It should be noted that in the study in Ghana the HIV status of the participants was not known, whilst in our study all participants were HIV-infected and receiving ART.

An earlier study of malaria prevalence conducted in HIV-positive pregnant females in Kigali, Rwanda, demonstrated that 8.0% of the study group had malaria [21]. It is well documented that pregnant women living in malaria endemic areas have an increased risk of *P. falciparum* infection during pregnancy but this usually remains asymptomatic. In the current study, we found seasonal fluctuation, with the prevalence of asymptomatic malaria being higher in subjects tested in the months of January–February than those tested in March–May.

In the current study, we report that pregnant females who were older than 30 years at the time of testing had a lower prevalence of helminth infections but higher levels of malaria than younger females. The helminth data is supported by a previous study from Uganda [30]; however, most studies show that malaria is also more common in younger, pregnant females [31], [32]. This difference may be related to a number of factors including lifestyle and socio-cultural differences across the population groups included in these studies.

Little data exists on the relationship between gravidity and the risk of helminthiasis. One study shows no effect of gravidity on the risk of helminth infection [28] whilst a second study demonstrates a higher risk of hookworm infection but a lower risk of *A. lumbricoides* infection in primigravid compared to multigravid females [32]. The data from the current study shows that primigravid females have a higher prevalence and risk of helminth infection compared to multigravid females. This is an important finding and suggests that de-worming programs should target such individuals. Our data also shows a higher fecal egg count in multigravid compared to primigravid females. Thus, although multigravid females are at a lower risk of helminth infections than primigravidae, when they do acquire a helminth infection they have a higher intensity of infection than primigravid females.

Females who presented for testing at an earlier stage of their pregnancy (4 months) had a higher prevalence of malaria and helminth-malaria co-infection than those at a later stage of pregnancy (5–6 months). This data is supported by findings from previous African studies [31], [33].

Education and employment acted as protective factors against both helminth infection and helminth-malaria co-infection. Previous studies have shown similar associations [28], [30], suggesting that socio-economic status is a strong modulator of disease risk.

Helminth infection was shown to be more prevalent in subjects who did not wash their hands. Studies have shown that the risk of helminth infection is reduced in subjects who regularly wash their hands, more so in those who use soap [34], [35]. Thus, simple changes in hygiene practices would be important for reducing the prevalence of helminth infections. In our analysis, however, hand washing was statistically significantly associated with an increased risk of malaria and – consequently - helminth-malaria co-infection. This finding is surprising and difficult to understand. Of note, the use of piped compared to river water reduced the risk of helminth infection but seemed to increase the risk for malaria. Whilst improved access to water is known to reduce the risk for helminth infection [36] the possible reasons for a greater risk of malaria associated with hand washing and piped water are not known, with little data available in the literature to confirm these associations. We believe that we are dealing here with a confounder, although it is apparently difficult to understand its nature, and neither an elevated social status nor local vector behavior or distribution offers any clue to understand this observation. However, one possible explanation is that stand pipes for the collection of water may have been situated in areas more suitable for mosquito breeding, or that puddle formation around stand pipes created favourable breeding conditions.

Helminth egg counts were highest in multigravidae who did not wear shoes regularly and who had low CD4 counts (Table 5). Hemoglobin levels were lowest in females who had helminth or malaria infections, who had low CD4 counts and who had a gestational age of 5 or 6 months. Based on the distinct mechanisms by which helminth and malaria affect hemoglobin levels, it can be speculated that their combined presence might interact to enhance the risk of anemia when intensity is moderately higher than in light worm intensities. The relationship between helminth infection, intensity and anemia has been described in several settings in Africa as well as in South East Asia [37]. Although women in our study group were all on ART with some having received nutritional supplements as part of their antenatal care package, previous regional studies also reported lower hemoglobin levels to be associated with high prevalence of helminth and malaria [38]. Our findings are further supported by other studies [17], [39] which report that pregnant women are known to exhibit fluctuating CD4 levels in pregnancy, which might expose them to higher helminth infection prevalence leading to maternal anemia. This could be explained by the fact that during pregnancy the immune system is impaired; therefore, HIV positive pregnant women who live in highly endemic areas in sub-Saharan Africa are likely to be at increased risk for helminth-malaria co-infections.

In the present study the risk of helminth infection was higher in females with a reduced CD4 cell count, and in subjects with a detectable viral load. This is in agreement with previous studies conducted in pregnant females in Uganda [38] and Rwanda [21] where CD4 counts correlated negatively with the risk of helminth infection. However, another study has found the opposite [38], although this investigation was not carried out in pregnant females. Webb et al. [40] reviewed the epidemiology and immunology of helminth–HIV interactions, and concluded that there is inconsistent data support to postulate a beneficial effect of anti-helminthic therapy on CD4 counts and viral load in HIV-1 co-infected individuals. With regard to malaria, we found that a low CD4 count was associated with an increased number of malaria episodes. This is in contrast with data from a very similar study performed in Rwanda, where no such an association was found [21]. This discrepancy may be related to the lower power of the earlier investigation. There is clear evidence from a number of studies that HIV does lead to more, and to more severe malaria episodes, particularly in pregnant women [41].

The prevalence of helminth infection was increased in subjects with malaria, and *vice versa*. A study conducted in Ghana on pregnant females also showed that helminth infection increased the risk of malaria [28]. It is thought that helminth infections have a number of effects on the immune system that leads to increased susceptibility to malaria [39], [42].

In our study population all subjects were taking ART irrespective of CD4 counts, as prescribed by the new (2010) Rwandan Ministry of Health guidelines for the prevention of mother to child transmission of HIV [25]. Helminth infections of any type, malaria or co-infection, were all less prevalent in subjects receiving AZT-3TC-NVP when compared to the other three ART regimens (Table 2). These effects remained significant for helminth infections after adjusting for confounding variables in a logistic regression analysis (Table 3). However, the protective effect of AZT-3TC-NVP for malaria was not sustained in the logistic regression model (Table 4). A previous study performed in pregnant Rwanda females also demonstrated that specific ART regimens seemed to reduce helminths prevalence but had less effect on malaria [21]; in this study the AZT-3TC-NVP regimen was the least protective compared with the other therapies i.e. AZT-NVP, d4T-3TC-NVP and AZT. This may be related to a much smaller sample size (n = 328) compared to the current study (n = 980), and the lack of data for confounding variables. Whilst these findings suggest a possible anti-helminthic effect of (certain) ART combinations, there was no non-ART control arm in both studies, as they were not designed to detect ART effects on helminth infections in the first place. Whilst there is therefore a limit to the interpretation of this finding, it warrants further investigation. Although the ART-induced reconstitution of cellular immunity would probably be the main factor for reducing helminth infections among HIV patients, previous *in vitro* and *in vivo* investigations indicated that HIV treatment, especially with protease inhibitors (PIs), could have a direct effect in killing of parasites including malaria [43]. It has been described before that ART without PIs may reduce the prevalence of helminth infection [21], [44]. Thus, ART itself might have contributed to the decline in helminth prevalence, asymptomatic malaria or co-infection seen in our study. We hypothesize that anti-mitochondrial toxicity of ART compounds may play a direct role here, a hypothesis for which support seems to accrue in a currently ongoing field trial in Gabon, designed to address this question (MP Grobusch and S Janssen, unpublished data).

The strengths of this study are the screening of a large number of women from eight health centers which caters for women of all socio-economic classes; the screening of three stool samples on three consecutive days for the presence of helminths; the use of a combination of two different screening techniques to increase the sensitivity of helminth diagnosis. However, our study has limitations. Methodologically, its cross-sectional design makes it impossible to determine temporal causality, including the inability of multivariate models to adjust for all confounding factors. All participants in the study were women and as hemoglobin levels differ between men and women, our findings cannot be extrapolated to men. The Kato Katz method used to determine the number of helminth eggs could have underestimated the proportion of women with light hookworm infection. The study had no control group of HIV-positive subjects not receiving ART.

In conclusion, we found that the prevalence of helminth infections, malaria and co-infections is common in HIV-positive pregnant women on ART in Rwanda. Helminth and malaria infection in this population are important risk factors for low hemoglobin levels. Subjects with low CD4 counts were at higher risk of infections and helminth infection is a risk factor for malaria. Education and employment were independent protective factors for helminth infection and malaria, whilst hand washing reduced the risk only for helminth infections. The possible antihelminthic effect of some ART combinations warrants further studies.
